# Global Stability of Delayed Viral Infection Models with Nonlinear Antibody and CTL Immune Responses and General Incidence Rate

**DOI:** 10.1155/2016/3903726

**Published:** 2016-12-15

**Authors:** Hui Miao, Zhidong Teng, Zhiming Li

**Affiliations:** College of Mathematics and System Sciences, Xinjiang University, Xinjiang, Urumqi 830046, China

## Abstract

The dynamical behaviors for a five-dimensional viral infection model with three delays which describes the interactions of antibody, cytotoxic T-lymphocyte (CTL) immune responses, and nonlinear incidence rate are investigated. The threshold values for viral infection, antibody response, CTL immune response, CTL immune competition, and antibody competition, respectively, are established. Under certain assumptions, the threshold value conditions on the global stability of the infection-free, immune-free, antibody response, CTL immune response, and interior equilibria are proved by using the Lyapunov functionals method, respectively. Immune delay as a bifurcation parameter is further investigated. The numerical simulations are performed in order to illustrate the dynamical behavior of the model.

## 1. Introduction

In recent years, many authors have formulated and studied mathematical models which describe the dynamics of virus population in vivo. These provide insights in our understanding of HIV (human immunodeficiency virus) and other viruses, such as HBV (hepatitis B virus) and HCV (hepatitis C virus) [1–34]. In particular, the global stability of steady states for these models will give us a detailed information and enhance our understanding about the viral dynamics.

During viral infections, the immune system reacts against virus. The antibody and CTL play the crucial roles in preventing and modulating infections. The antibody response is implemented by the functioning of immunocompetent B lymphocytes. The CTL immune response has the ability to suppress the virus replication in vivo. Hence, in order to prevent virus infection, an effective vaccine needs both strong neutralizing antibody and CTL immune responses [1, 2, 14, 18–23, 25–32]. Based on these, it is of interest for us to investigate whether sustained oscillations are the result of delayed viral infection model. This provides us with the motivation to conduct our work. In [2], Balasubramaniam et al. developed the viral infection model by incorporating immune delays and Beddington-DeAngelis incidence rate

(1) dxtdt=λ−dxt−β1−ϵrtxtvt1+mxt+nvt,dytdt=β1−ϵrtxtvt1+mxt+nvt−ayt−pytzt,dvtdt=k1−ϵpiyt−uvt−qvtwt,dwtdt=gvtwt−hwt,dztdt=cyt−τzt−τ−bzt,

where *x*, *y*, *v*, *w*, and *z* denote the concentrations of susceptible host cells, infected cells, free virus, antibody responses, and CTL immune responses, respectively. The local and global stability of the infection-free equilibrium and infected equilibrium and the existence of Hopf bifurcation are obtained. Furthermore, by using the Nyquist criterion, the estimation of the length of the delay to preserve stability of the infected equilibrium is obtained.

Motivated by the work in [1, 2, 20, 21], in the present paper we propose a general viral infection model with three time delays which describes the interactions of antibody, CTL immune responses, and nonlinear incidence rate

(2) dxtdt=sx−fx,v,dytdt=e−m1τ1fxt−τ1,vt−τ1−ag1y−pg1yg4z,dvtdt=ke−m2τ2g1yt−τ2−ug2v−qg2vg3w,dztdt=cg1yt−τ3g4zt−τ3−bg4z,dwtdt=rg2vg3w−hg3w,

where *s*(*x*) denotes the intrinsic growth rate of uninfected target cells accounting for both production and natural mortality. In the literature of virus dynamics, the typical forms of the growth rate are *s*(*x*) = *λ* − *dx* and *s*(*x*) = *λ* − *dx* + *rx*(1 − *x*/*K*), where *λ*, *d*, *r*, *K* are positive real numbers [4–13, 15, 16, 18, 20–23, 26–32, 34].

We assume that the incidence of new infections of target cells occurs at a rate *f*(*x*, *v*). This form of incident rate is general to encompass several forms such as bilinear incidence *βxv* [4, 13], saturated incidence *βxv*/(1 + *bv*) [16], Holling type II functional response *βxv*/(1 + *ax*) [15], and Crowley-Martin incidence *βxv*/(1 + *ax* + *bv* + *abxv*) [12, 35], where *β*, *a*, and *b* are positive constants.

It is also assumed that the death rates of the infected target cells, viruses, antibody, and CTLs depend on their concentrations. These rates are given by *ag*_1_(*y*), *ug*_2_(*v*), *hg*_3_(*w*), and *bg*_4_(*z*), respectively. The neutralization rate of viruses and the activation rate of B cells are proportional to the product of the removal rates of the viruses and B cells. Let *qg*_2_(*v*)*g*_3_(*w*) and *rg*_2_(*v*)*g*_3_(*w*) be the neutralization rate of viruses and activation rate of B cells, respectively. The typical forms can be seen as *qvw* and *rvw* [1, 2, 20, 21, 31, 32]. Accordingly, let *pg*_1_(*y*)*g*_4_(*z*) and *cg*_1_(*y*)*g*_4_(*z*) be the killing rate of infected cells and the birth rate of the CTL cells, respectively. The typical forms are *pyz* and *cyz* that appear in several papers [1, 2, 14, 20, 22, 27, 30, 34].

For model (2), based on the epidemiological background, we assume that virus production occurs after the virus entry by the time delay *τ*_1_. The probability of surviving the time period from *t* − *τ*_1_ to *t* is *e* ^−*m*_1_*τ*_1_^. Let *τ*_2_ be the maturation time of the newly produced viruses. The constant *e* ^−*m*_2_*τ*_2_^ denotes the surviving rate of virus during the delay period. Antigenic stimulation generating CTL cell may need a period of time *τ*_3_.

In this paper, our purpose is to investigate the dynamical properties of model (2), including the local and global stability of equilibria. The reproduction numbers for viral infection, antibody response, CTL immune response, CTL immune competition, and antibody competition, respectively, are calculated. By using Lyapunov functionals and LaSalle's invariance principle, the threshold conditions for the global asymptotic stability of infection-free equilibrium *E*_0_, immune-free equilibrium *E*_1_, infection equilibrium *E*_2_ only with antibody response, and infection equilibrium *E*_3_ only with CTL immune response and infection equilibrium *E*_4_ with both antibody and CTL immune responses when the delay *τ*_3_ = 0, respectively, are established. By using the linearization method, the instability of equilibria *E*_0_, *E*_1_, *E*_2_, and *E*_3_, respectively, is also established. Furthermore, by using the numerical simulation method, we will discuss the existence of the Hopf bifurcation and stability switches at equilibria *E*_3_ and *E*_4_ when *τ*_3_ > 0.

The organization of this paper is as follows. In the next section, the basic properties of model (2) for the positivity and boundedness of solutions, the threshold values, and the existence of equilibria are discussed. In Section 3, the threshold conditions on the global stability and instability of equilibria *E*_0_, *E*_1_, and *E*_2_ are proved. When *τ*_3_ = 0, the threshold conditions on the global stability and instability for equilibria *E*_3_ and *E*_4_ are stated and proved. In Section 4, the numerical simulations are given to further discuss the stability of equilibria *E*_3_ and *E*_4_ when *τ*_3_ > 0. It is shown that the Hopf bifurcation and stability switches at these equilibria occur as *τ*_3_ increases. In the last section, we offer a brief conclusion.

## 2. Preliminaries

Let *τ* = max⁡{*τ*_1_, *τ*_2_, *τ*_3_} and *R*_+_^5^ = {(*x*_1_, *x*_2_, *x*_3_, *x*_4_, *x*_5_) : *x*_*i*_ ≥ 0, *i* = 1,2,…, 5}. *C*([−*τ*, 0], *R*_+_^5^) denotes the space of continuous functions mapping interval [−*τ*, 0] into *R*_+_^5^ with norm ‖*ϕ*‖ = sup_−*τ*≤*t*≤0_⁡{|*ϕ*(*t*)|} for any *ϕ* ∈ *C*([−*τ*, 0], *R*_+_^5^).

The initial conditions for any solutions of model (2) are given as follows:

(3) xθ,yθ,vθ,zθ,wθ=ϕ1θ,ϕ2θ,ϕ3θ,ϕ4θ,ϕ5θ,ϕiθ≥0,θ∈−τ,0,  ϕi0>0,  i=1,2,3,4,5,

where (*ϕ*_1_(*θ*), *ϕ*_2_(*θ*), *ϕ*_3_(*θ*), *ϕ*_4_(*θ*), *ϕ*_5_(*θ*)) ∈ *C*([−*τ*, 0], *R*_+_^5^). By the fundamental theory of functional differential equation [36], model (2) admits a unique solution (*x*(*t*), *y*(*t*), *v*(*t*), *z*(*t*), *w*(*t*)) satisfying initial conditions (3).

In this paper, we firstly introduce the following assumptions:

(*H*_1_) *s*(*x*) is continuously differentiable. There exists $\bar{x}>0$ such that $s(\bar{x})=0$ and $s^{\prime }(\bar{x})<0$.
(*H*_2_) *f*(*x*, *v*) is continuously differentiable; *f*(*x*, *v*) > 0 for *x* ∈ (0, *∞*), *v* ∈ (0, *∞*); *f*(*x*, *v*) = 0 if and only if *x* = 0 or *v* = 0; ∂*f*(*x*, *v*)/∂*x* ≥ 0 and ∂*f*(*x*, *v*)/∂*v* ≥ 0 for all *x* ≥ 0 and *v* ≥ 0; (*d*/*dx*)(∂*f*(*x*, 0)/∂*v*) ≥ 0 for all *x* ≥ 0.
(*H*_3_) *g*_*i*_(*ξ*)  (*i* = 1,2, 3,4) is strictly increasing on [0, *∞*); lim_*ξ*→*∞*_⁡ *g*_*i*_(*ξ*) = +*∞*; and there exists *k*_*i*_ > 0 such that *g*_*i*_(*ξ*) ≥ *k*_*i*_ *ξ* for any *ξ* ≥ 0; *g*_*i*_(0) = 0 and *g*_*i*_′(0) = 1.
(*H*_4_) *f*(*x*, *v*)/*g*_2_(*v*) is nonincreasing with respect to *v* for *v* ∈ (0, *∞*).

From (*H*_1_) we easily obtain that *s*(*x*) > 0 for all $0<x<\bar{x}$ and *s*(*x*) < 0 for all $x>\bar{x}$. Assumption (*H*_1_) shows that the number of healthy cells *x* has a maximum capacity $\bar{x}$ in the absence of infection. When $x<\bar{x}$, *s*(*x*) has a positive growth; if $x>\bar{x}$ it has a negative growth. Assumption (*H*_2_) implies that there are no new infected cells (i.e., *f*(*x*, *v*) = 0) without healthy cells (*x* = 0) or virus (*v* = 0). The higher the number of healthy cells *x* is, the higher the number of healthy cells *x* which are infected in the unit time will be. Similarly, the higher the amount of virus *v* is, the higher the number of healthy cells *x* which are infected in the unit time will be. Assumption (*H*_3_) assumes that the death rates of the infected target cells *y*, virus *v*, antibodies *w*, and CTLs *z* depend on their concentrations. If these numbers *y*, *v*, *w*, *z* increase, the corresponding rates *ag*_1_(*y*), *ug*_2_(*v*), *hg*_3_(*w*), and *bg*_4_(*z*) will increase, and the ratio *g*_*i*_(*ξ*)/*ξ* is no less than a positive constant for *i* = 1,2, 3,4. Finally, assumption (*H*_4_) indicates that both the rate of new infections of target cells and the virus clearance rate increase according to the level of virus. However, the corresponding ratio is nonincreasing.

Using an argument similar to [14] we have the following result.

**Theorem 1 .**

Assume that (*H*_1_)–(*H*_4_) hold. Let (*x*(*t*), *y*(*t*), *v*(*t*), *z*(*t*), *w*(*t*)) be the solution of model (2) with initial conditions (3); then (*x*(*t*), *y*(*t*), *v*(*t*), *z*(*t*), *w*(*t*)) is positive and ultimately bounded.

Next, we discuss the existence and uniqueness of equilibria of model (2). We know that any equilibrium *E* = (*x*, *y*, *v*, *z*, *w*) of model (2) satisfies

(4) sx−fx,v=0,e−m1τ1fx,v−ag1y−pg1yg4z=0,ke−m2τ2g1y−ug2v−qg2vg3w=0,cg1yg4z−bg4z=0,rg2vg3w−hg3w=0.

It is clear from (4) that model (2) has a unique infection-free equilibrium $E_{0}=(\bar{x},0,0,0,0)$. When *y* = 0, from (4) we have *s*(*x*) = *f*(*x*, *v*), *g*_2_(*v*)(*u* + *qg*_3_(*w*)) = 0, *g*_4_(*z*) = 0, and (*rg*_2_(*v*) − *h*)*g*_3_(*w*) = 0. Solving these equations, we have $x=\bar{x}$, *v* = 0, *z* = 0, and *w* = 0. When *v* = 0, from (4) we have *s*(*x*) = 0, *g*_1_(*y*)(*a* + *pg*_4_(*z*)) = 0, *g*_1_(*y*) = 0, *g*_4_(*z*) = 0, and *g*_3_(*w*) = 0. Solving these equations, we have $x=\bar{x}$, *v* = 0, *z* = 0, and *w* = 0. Therefore, besides equilibrium *E*_0_, model (2) only has the following four possible equilibria: *E*_1_ = (*x*_1_, *y*_1_, *v*_1_, 0,0), *E*_2_ = (*x*_2_, y_2_, *v*_2_, 0, *w*_2_), *E*_3_ = (*x*_3_, *y*_3_, *v*_3_, *z*_3_, 0), and *E*_4_ = (*x*_4_, *y*_4_, *v*_4_, *z*_4_, *w*_4_).

The existence of immune-free equilibrium *E*_1_ = (*x*_1_, *y*_1_, *v*_1_, 0,0) is equivalent to the existence of positive solution (*x*_1_, *y*_1_, *v*_1_) of the following equations:

(5) $$\begin{array}{c} s\left(\;x\;\right)=f\left(\;x,v\;\right)=ae^{m_{1}\tau_{1}}g_{1}\left(\;y\;\right)=\frac{aue^{m_{1}\tau_{1}+m_{2}\tau_{2}}}{k}g_{2}\left(\;v\;\right). \end{array}$$

By (*H*_3_), the inverse function *g*_2_^−1^(*v*) exists. Solving *s*(*x*) = (*aue* ^*m*_1_*τ*_1_+*m*_2_*τ*_2_^/*k*)*g*_2_(*v*), we have *v* = *φ*(*x*)≜*g*_2_^−1^(*ks*(*x*)/*aue* ^*m*_1_*τ*_1_+*m*_2_*τ*_2_^) with $\varphi (\bar{x})=0$ and *φ*(0) = *v* ^0^, where *v* ^0^ is the unique positive root of equation *s*(0) = (*aue* ^*m*_1_*τ*_1_+*m*_2_*τ*_2_^/*k*)*g*_2_(*v*). Define *G*(*x*) = *f*(*x*, *φ*(*x*))−(*aue* ^*m*_1_*τ*_1_+*m*_2_*τ*_2_^/*k*)*g*_2_(*φ*(*x*)). Then *G*(0) = −(*aue* ^*m*_1_*τ*_1_+*m*_2_*τ*_2_^/*k*)*g*_2_(*v* ^0^) < 0 and $G(\bar{x})=0.$

Define the basic reproduction number for viral infection

(6) $$\begin{array}{c} R_{0}=\frac{ke^{-m_{1}\tau_{1}-m_{2}\tau_{2}}}{au}\frac{\partial f\left(\bar{x},0\right)}{\partial v}. \end{array}$$

Note that

(7) G′x¯=∂fx¯,0∂x+∂fx¯,0∂vφ′x¯−auem1τ1+m2τ2kg2′0φ′x¯=auem1τ1+m2τ2kφ′x¯kauem1τ1+m2τ2∂fx¯,0∂v−1=s′x¯R0−1.

Thus, if *R*_0_ > 1, then $G^{\prime }(\bar{x})<0.$ This implies that there exists $x_{1}\in (0,\bar{x})$ such that *G*(*x*_1_) = 0. The value of *v*_1_ is given by *v*_1_ = *φ*(*x*_1_). (*H*_3_) ensures that *ke* ^−*m*_2_*τ*_2_^ *g*_1_(*y*) = *ug*_2_(*v*_1_) has a unique positive solution *y*_1_ = *g*_1_^−1^(*ue* ^*m*_2_*τ*_2_^ *g*_2_(*v*_1_)/*k*). Therefore, *E*_1_ exists if *R*_0_ > 1.

Next we show that *E*_1_ = (*x*_1_, *y*_1_, *v*_1_, 0,0) is a unique immune-free equilibrium. Otherwise, there exists another *E*_1_^*∗*^ = (*x*_1_^*∗*^, *y*_1_^*∗*^, *v*_1_^*∗*^, 0,0). Without of loss of generality, we assume that *x*_1_^*∗*^ < *x*_1_, and then *s*(*x*_1_^*∗*^) > *s*(*x*_1_). Meanwhile, *ks*(*x*_1_) = *aue* ^*m*_1_*τ*_1_+*m*_2_*τ*_2_^ *g*_2_(*v*_1_) and *ks*(*x*_1_^*∗*^) = *aue* ^*m*_1_*τ*_1_+*m*_2_*τ*_2_^ *g*_2_(*v*_1_^*∗*^). By (*H*_3_) and (*H*_4_), we have *v*_1_^*∗*^ > *v*_1_ and *f*(*x*_1_, *v*_1_^*∗*^)/*g*_2_(*v*_1_^*∗*^) ≤ *f*(*x*_1_, *v*_1_)/*g*_2_(*v*_1_). Since *x*_1_^*∗*^ < *x*_1_, we obtain *f*(*x*_1_, *v*_1_^*∗*^) > *f*(*x*_1_^*∗*^, *v*_1_^*∗*^) and *f*(*x*_1_^*∗*^, *v*_1_^*∗*^)/*g*_2_(*v*_1_^*∗*^) < *f*(*x*_1_, *v*_1_)/*g*_2_(*v*_1_). For another, we have *f*(*x*_1_^*∗*^, *v*_1_^*∗*^)/*g*_2_(*v*_1_^*∗*^) = *f*(*x*_1_, *v*_1_)/*g*_2_(*v*_1_). This is a contradiction. Thus *E*_1_ is a unique equilibrium.

We consider the existence of infection equilibrium *E*_2_ = (*x*_2_, *y*_2_, *v*_2_, 0, *w*_2_) with only antibody response. It is clear that *v*_2_ = *g*_2_^−1^(*h*/*r*). Define *F*(*x*) = *s*(*x*) − *f*(*x*, *v*_2_). By (*H*_1_) and (*H*_2_), we obtain *F*′(*x*) < 0. Since *F*(0) = *s*(0) > 0 and $F(\bar{x})=s(\bar{x})-f(\bar{x},v_{2})<0$, there exists a unique $x_{2}\in (0,\bar{x})$ such that *F*(*x*_2_) = 0. Then, we have *y*_2_ = *g*_1_^−1^(*e* ^−*m*_1_*τ*_1_^ *f*(*x*_2_, *v*_2_)/*a*).

Define the constant

(8) $$\begin{array}{c} R_{1}=\frac{ke^{-m_{1}\tau_{1}-m_{2}\tau_{2}}}{u}\frac{f\left(x_{2},v_{2}\right)}{g_{2}\left(v_{2}\right)}, \end{array}$$

which is called the antibody response reproductive number of model (2). Solving *w*_2_ from (4), we obtain that

(9) w2g3−1ke−m2τ2g1y2−ug2v2qg2v2=g3−1uR1−1q>0if  R1>1.

Therefore, *E*_2_ exists and is unique if *R*_1_ > 1.

We consider the existence of infection equilibrium *E*_3_ = (*x*_3_, *y*_3_, *v*_3_, *z*_3_, 0) with only CTL immune response. From the third and fourth equations of (4), we obtain unique *y*_3_ = *g*_1_^−1^(*b*/*c*) and v_3_ = *g*_2_^−1^(*bke* ^−*m*_2_*τ*_2_^/*cu*). Define *F*(*x*) = *s*(*x*) − *f*(*x*, *v*_3_). By (*H*_1_) and (*H*_2_), we obtain *F*′(*x*) < 0. Since *F*(0) = *s*(0) > 0 and $F(\bar{x})=s(\bar{x})-f(\bar{x},v_{3})<0$, there exists a unique $x_{3}\in (0,\bar{x})$ such that *F*(*x*_3_) = 0.

Define the constant

(10) $$\begin{array}{c} R_{2}=\frac{ke^{-m_{1}\tau_{1}-m_{2}\tau_{2}}}{au}\frac{f\left(x_{3},v_{3}\right)}{g_{2}\left(v_{3}\right)}, \end{array}$$

which is called the CTL immune response reproductive number of model (2). Solving the second equation for *z* yields

(11) z3g4−1e−m1τ1fx3,v3−ag1y3pg1y3=g4−1aR2−1p>0if  R2>1.

Therefore, *E*_3_ exists and is unique if *R*_2_ > 1.

Lastly, we consider the existence of infection equilibrium *E*_4_ = (*x*_4_, *y*_4_, *v*_4_, *z*_4_, *w*_4_) with both antibody and CTL immune responses. From the fourth and fifth equation of (4), we obtain unique *y*_4_ = *g*_1_^−1^(*b*/*c*) and *v*_4_ = *g*_2_^−1^(*h*/*r*). Define *F*(*x*) = *s*(*x*) − *f*(*x*, *v*_4_). By (*H*_1_) and (*H*_2_), we obtain *F*′(*x*) < 0. Since *F*(0) = *s*(0) > 0 and $F(\bar{x})=s(\bar{x})-f(\bar{x},v_{4})<0$, there exists a unique $x_{4}\in (0,\bar{x})$ such that *F*(*x*_4_) = 0.

Define the constants

(12) R3=cfx4,v4abem1τ1,R4=kbruchem2τ2,

which are called the CTL immune response competitive reproductive number and the antibody response competitive reproductive number of model (2), respectively. Solving the second equation for *z* yields a unique

(13) z4g4−1e−m1τ1fx4,v4−ag1y4pg1y4=g4−1aR3−1p>0if  R3>1.

Solving the third equation for *w*, we further obtain a unique

(14) w4g3−1ke−m2τ2g1y4−ug2v4qg2v4=g3−1uR4−1q>0if  R4>1.

Therefore, *E*_4_ exists and is unique if *R*_3_ > 1 and *R*_4_ > 1.

**Remark 2 .**

From (*H*_2_) and (*H*_4_), we obtain *R*_1_ < *R*_0_ and *R*_2_ < *R*_0_. In fact,

(15) R1ke−m1τ1−m2τ2ufx2,v2gv2≤ke−m1τ1−m2τ2ulimv→0+⁡fx2,vg2v=ke−m1τ1−m2τ2ug2′0∂fx2,0∂v<ke−m1τ1−m2τ2ug2′0∂fx¯,0∂v=R0,R2ke−m1τ1−m2τ2aufx3,v3g2v3≤ke−m1τ1−m2τ2aulimv→0+⁡fx3,vg2v=ke−m1τ1−m2τ2aug2′0∂fx3,0∂v<ke−m1τ1−m2τ2aug2′0∂fx¯,0∂v=R0.

## 3. Stability Analysis

### 3.1. Stability of Equilibrium *E*_0_

**Theorem 3 .**

(a) If *R*_0_ ≤ 1, then infection-free equilibrium *E*_0_ is globally asymptotically stable.

(b) If *R*_0_ > 1, then *E*_0_ is unstable.

**Proof**

Consider conclusion (a). Define a Lyapunov functional *V*_1_(*t*) as follows:

(16) V1t=xt−∫x¯xtlimv→0⁡fx¯,vfθ,v dθ+em1τ1yt+aem1τ1+m2τ2kvt+pem1τ1czt+∫−τ10fxt+s,vt+sds+aem1τ1∫−τ20g1yt+sds+pem1τ1∫−τ30g1yt+sg4zt+sds+aqem1τ1+m2τ2krwt.

Calculating the time derivative of *V*_1_(*t*) along solutions of model (2), we obtain

(17) dV1tdt=sx1−limv→0⁡fx¯,vfx,v+fx,v·limv→0⁡fx¯,vfx,v−auem1τ1+m2τ2kg2v−pbem1τ1c·g4z−aqhem1τ1+m2τ2krg3w≤auem1τ1+m2τ2k·g2vkauem1τ1+m2τ2fx,vg2vlimv→0⁡fx¯,vfx,v−1+sx1−limv→0⁡fx¯,vfx,v.

Note that $s(x)(1-\lim_{v\to 0}\;(f\left(\bar{x},v\right)/f\left(x,v\right)))\leq 0$, and

(18) fx,vg2vlimv→0⁡fx¯,vfx,vlimv→0⁡fx,vg2v∂fx¯,0/∂v∂fx,0/∂v=∂fx¯,0∂v1g2′0.

It follows that

(19) $$\begin{array}{c} \frac{dV_{1}\left(t\right)}{dt}\leq \frac{aue^{m_{1}\tau_{1}+m_{2}\tau_{2}}}{k}g_{2}\left(\;v\;\right)\left(\;R_{0}-1\;\right). \end{array}$$

Note that *dV*_1_(*t*)/*dt* = 0 if and only if $x(t)=\bar{x}$, *v*(*t*) = 0, *z*(*t*) = 0, *y*(*t*) = 0, and *w*(*t*) = 0. So, the maximal compact invariant set in {(*x*, *y*, *v*, *z*, *w*) ∈ *R*_+_^5^ : *dV*_1_(*t*)/*dt* = 0} is singleton {*E*_0_}. By LaSalle's invariance principle [36], *E*_0_ is globally asymptotically stable.

Next, we consider conclusion (b). By computing, the characteristic equation of the linearization system of model (2) at *E*_0_ is

(20) $$\begin{array}{c} \left(\;\lambda +hg_{3}^{\prime }\left(\;0\;\right)\;\right)\left(\;\lambda +bg_{4}^{\prime }\left(\;0\;\right)\;\right)\left(\;\lambda -s^{\prime }\left(\;\bar{x}\;\right)\;\right)f\left(\;\lambda \;\right)=0, \end{array}$$

where

(21) fλ=λ2+a+uλ+au−k∂fx¯,0∂ve−m1+λτ1e−m2+λτ2.

When *R*_0_ > 1, we have $f(0)=au-k(\partial f\left(\bar{x},0\right)/\partial v)e^{-m_{1}\tau_{1}}e^{-m_{2}\tau_{2}}<0$ and lim_*λ*→+*∞*_⁡ *f*(*λ*) = +*∞*. Hence, there is *λ* > 0 such that *f*(*λ*) = 0. Therefore, when *R*_0_ > 1, *E*_0_ is unstable. This completes the proof.

**Remark 4 .**

Theorem 3 shows that if only equilibrium *E*_0_ exists, then it is globally asymptotically stable, and delays *τ*_1_, *τ*_2_, and *τ*_3_ do not impact the stability of *E*_0_.

### 3.2. Stability of Equilibrium *E*_1_

Firstly, we introduce two lemmas which will be used in the proof of Theorem 7.

**Lemma 5 .**

Suppose that (*H*_1_)–(*H*_4_) hold and *R*_0_ > 1. Let *x*_2_ and *v*_2_ satisfy *g*_2_(*v*_2_) = *h*/*r* and *s*(*x*_2_) = *f*(*x*_2_, *v*_2_). Then for equilibrium *E*_1_ = (*x*_1_, *y*_1_, *v*_1_, 0,0), sign⁡(*x*_2_ − *x*_1_) = sign⁡(*v*_1_ − *v*_2_) = sign⁡(*R*_1_ − 1).

**Proof**

Since *s*(*x*_1_) = *f*(*x*_1_, *v*_1_), we have

(22) sx2−sx1=fx2,v2−fx1,v2+fx1,v2−fx1,v1.

By (*H*_1_) and (*H*_2_), we get sign⁡(*x*_2_ − *x*_1_) = sign⁡(*v*_1_ − *v*_2_). Using (*ke* ^−*m*_1_*τ*_1_−*m*_2_*τ*_2_^/*au*)(*f*(*x*_1_, *v*_1_)/*g*_2_(*v*_1_)) = 1, we have

(23) R1−1=kauem1τ1+m2τ21g2v2fx2,v2−fx1,v2+fx1,v2g2v2−fx1,v1g2v1.

By (*H*_2_) and (*H*_4_), it follows that sign⁡(*R*_1_ − 1) = sign⁡(*v*_1_ − *v*_2_). This completes the proof.

**Lemma 6 .**

Suppose that (*H*_1_)–(*H*_4_) hold and *R*_0_ > 1. Let *x*_3_, *y*_3_, and *v*_3_ satisfy *g*_2_(*v*_3_) = *kbe* ^−*m*_2_*τ*_2_^/*uc*, *g*_1_(*y*_3_) = *b*/*c*, and *s*(*x*_3_) = *f*(*x*_3_, *v*_3_). Then, for equilibrium *E*_1_ = (*x*_1_, *y*_1_, *v*_1_, 0,0), sign⁡(*x*_3_ − *x*_1_) = sign⁡(*v*_1_ − *v*_3_) = sign⁡(*y*_1_ − *y*_3_) = sign⁡(*R*_2_ − 1).

**Proof**

Since *g*_1_(*y*_1_) = (*ue* ^*m*_2_*τ*_2_^/*k*)*g*_2_(*v*_1_) and *g*_1_(*y*_3_) = (*ue* ^*m*_2_*τ*_2_^/*k*)*g*_2_(*v*_3_), we have sign⁡(*v*_1_ − *v*_3_) = sign⁡(*y*_1_ − *y*_3_). Since *s*(*x*_1_) = *f*(*x*_1_, *v*_1_), one has

(24) sx3−sx1=fx3,v3−fx3,v1+fx3,v1−fx1,v1.

By (*H*_1_) and (*H*_2_), we get sign⁡(*x*_3_ − *x*_1_) = sign⁡(*v*_1_ − *v*_3_), and

(25) R2−1=kauem1τ1+m2τ2fx3,v3g2v3−fx3,v1g2v1+fx3,v1−fx1,v1g2v1.

By (*H*_2_) and (*H*_4_), we further have sign⁡(*R*_2_ − 1) = sign⁡(*x*_3_ − *x*_1_). This completes the proof.

**Theorem 7 .**

Let *R*_0_ > 1. (a) If *R*_1_ ≤ 1 and *R*_2_ ≤ 1, then immune-free equilibrium *E*_1_ is globally asymptotically stable. (b) If *R*_1_ > 1 or *R*_2_ > 1, then *E*_1_ is unstable.

**Proof**

Consider conclusion (a). Denote *H*(*ξ*) = *ξ* − 1 − ln⁡*ξ* with *ξ* ∈ *R*_+_. Define a Lyapunov functional *V*_2_(*t*) as follows:

(26) V2t=xt−∫x1xtfx1,v1fθ,v1 dθ+em1τ1yt−∫y1ytg1y1g1θ dθ+aem1τ1+m2τ2kvt−∫v1vtg2v1g2θ dθ+pem1τ1czt+aqem1τ1+m2τ2krwt+fx1,v1∫−τ10Hfxt+s,vt+sfx1,v1ds+pem1τ1∫−τ30g1yt+sg4zt+sds+aem1τ1g1y1∫−τ20Hg1yt+sg1y1ds.

Calculating the derivative of *V*_2_(*t*) along solutions of model (2), we obtain

(27) dV2tdt=sx1−fx1,v1fx,v1+fx,vfx1,v1fx,v1−auem1τ1+m2τ2kg2v+M1+M2,

where

(28) M1=pem1τ1g1y1g4z−pbem1τ1cg4z+aqem1τ1+m2τ2kg2v1g3w−aqhem1τ1+m2τ2kr·g3w=pem1τ1g4zg1y1−g1y3+aqem1τ1+m2τ2kg3wg2v1−g2v2,M2=fx1,v12−g1y1fxt−τ1,vt−τ1g1yfx1,v1−g2v1g1yt−τ2g1y1g2v+ln⁡fxt−τ1,vt−τ1fx,v+ln⁡g1yt−τ2g1y=fx1,v1·ln⁡g2vfx1,v1g2v1fx,v−fx1,v1·Hg1y1fxt−τ1,vt−τ1g1yfx1,v1−fx1,v1Hg2v1g1yt−τ2g1y1g2v.

Therefore,

(29) dV2tdt=sx−sx11−fx1,v1fx,v1−fx1,v1Hg2v1g1yt−τ2g1y1g2v+fx1,v1g2vg2v1fx,vfx,v1−1·g2v1g2v−fx,v1fx,v+M1−fx1,v1·Hfx1,v1fx,v1−fx1,v1·Hg2vfx,v1g2v1fx,v−fx1,v1·Hg1y1fxt−τ1,vt−τ1g1yfx1,v1.

Note that (*s*(*x*) − *s*(*x*_1_))(1 − *f*(*x*_1_, *v*_1_)/*f*(*x*, *v*_1_)) ≤ 0, and

(30) fx,vfx,v1−1g2v1g2v−fx,v1fx,v≤0for  t≥0.

Lemmas 5 and 6 imply that *y*_1_ ≤ *y*_3_ and *v*_1_ ≤ *v*_2_ if *R*_1_ ≤ 1 and *R*_2_ ≤ 1. It then follows from the monotonicity of *g*_1_ and *g*_2_ that *M*_1_ ≤ 0. We have *dV*_2_(*t*)/*dt* ≤ 0, and *dV*_2_(*t*)/*dt* = 0 if and only if *x*(*t*) = *x*_1_, *y*(*t*) = *y*_1_, *v*(*t*) = *v*_1_, *z*(*t*) = 0, and *w*(*t*) = 0. From LaSalle's invariance principle [36], we finally have that equilibrium *E*_1_ of model (2) is globally asymptotically stable when *R*_0_ > 1, *R*_1_ ≤ 1, and *R*_2_ ≤ 1.

Next, consider conclusion (b). By computing, the characteristic equation of the linearization system of model (2) at *E*_1_ is

(31) $$\begin{array}{c} \left(\;\lambda +h-rg_{2}\left(\;v_{1}\;\right)\;\right)f_{1}\left(\;\lambda \;\right)f_{2}\left(\;\lambda \;\right)=0, \end{array}$$

where *f*_1_(*λ*) = *λ* + *b* − *cg*_1_(*y*_1_)*e* ^−*λτ*_3_^ and

(32) $$\begin{array}{c} f_{2}\left(\;\lambda \;\right)=\left|\begin{array}{ccc} \lambda -s^{\prime }\left(x_{1}\right)+\frac{\partial f\left(x_{1},v_{1}\right)}{\partial x} & 0 & \frac{\partial f\left(x_{1},v_{1}\right)}{\partial v}\\ -e^{-\left(m_{1}+\lambda \right)\tau_{1}}\frac{\partial f\left(x_{1},v_{1}\right)}{\partial x} & \lambda +ag_{1}^{\prime }\left(y_{1}\right) & -e^{-\left(m_{1}+\lambda \right)\tau_{1}}\frac{\partial f\left(x_{1},v_{1}\right)}{\partial v}\\ 0 & -ke^{-\left(m_{2}+\lambda \right)\tau_{2}}g_{1}^{\prime }\left(y_{1}\right) & \lambda +ug_{2}^{\prime }\left(v_{1}\right) \end{array}\right|. \end{array}$$

When *R*_1_ > 1, we have *h* − *rg*_2_(*v*_1_) = *r*(*g*_2_(*v*_2_) − *g*_2_(*v*_1_)) < 0. Hence, there is a positive root *λ* ^*∗*^ = *rg*_2_(*v*_1_) − *h*. When *R*_2_ > 1, we have *f*_1_(0) = *b* − *cg*_1_(*y*_1_) = *c*(*g*_1_(*y*_3_) − *g*_1_(*y*_1_)) < 0 and lim_*λ*→+*∞*_⁡ *f*_1_(*λ*) = +*∞*. Hence, there is also a positive root *λ* ^*∗*^ such that *f*_1_(*λ* ^*∗*^) = 0. Therefore, when *R*_1_ > 1 or *R*_2_ > 1, *E*_1_ is unstable. This completes the proof.

**Remark 8 .**

Theorem 7 shows that if only equilibria *E*_0_ and *E*_1_ exist, then *E*_1_ is globally asymptotically stable, and delays *τ*_1_, *τ*_2_, and *τ*_3_ do not impact the stability of *E*_1_.

### 3.3. Stability of Equilibrium *E*_2_

We firstly have the following Lemma.

**Lemma 9 .**

Suppose *R*_1_ > 1 and *R*_3_ ≤ 1. Let $\bar{E_{4}}=(\bar{x_{4}},\bar{y_{4}},\bar{v_{4}},\bar{z_{4}},\bar{w_{4}})$ be the solution of equation (4) with $\bar{v_{4}}=g_{2}^{-1}(h/r)$ and $\bar{y_{4}}=g_{1}^{-1}(b/c).$ Then for equilibrium $E_{2}=(x_{2},y_{2},v_{2},0,w_{2}),y_{2}\leq \bar{y_{4}}$.

**Proof**

Since $\bar{E_{4}}$ satisfies (4), we have $\bar{y_{4}}=g_{1}^{-1}(b/c)$, $\bar{v_{4}}=g_{2}^{-1}(h/r)$, and $\bar{x_{4}}=x_{2}.$ Compared with *E*_4_, we obtain $\bar{x_{4}}=x_{4}$ and $\bar{v_{4}}=v_{4}.$ When *R*_3_ ≤ 1, we get $\bar{z_{4}}\leq 0.$ Since

(33) e−m1τ1fx2,v2=ag1y2,e−m1τ1fx4¯,v4¯=ag1y4¯+pg1y4¯g4z4¯,

it follows that $y_{2}\leq \bar{y_{4}}$ if *R*_1_ > 1 and *R*_3_ ≤ 1. This completes the proof.

**Theorem 10 .**

Let *R*_1_ > 1. (a) If *R*_3_ ≤ 1, then antibody response equilibrium *E*_2_ is globally asymptotically stable.

(b) If *R*_3_ > 1, then *E*_2_ is unstable.

**Proof**

Consider conclusion (a). Define a Lyapunov functional *V*_3_(*t*) as follows:

(34) V3t=xt−∫x2xtfx2,v2fθ,v2 dθ+em1τ1yt−∫y2ytg1y2g1θ dθ+fx2,v2g2v2u+qg3w2vt−∫v2vtg2v2g2θ dθ+pem1τ1czt+qfx2,v2g2v2ru+qg3w2wt−∫w2wtg3w2g3θ dθ+fx2,v2·∫−τ10Hfxt+s,vt+sfx2,v2ds+fx2,v2∫−τ20Hg1yt+sg1y2ds+pem1τ1∫−τ30g1yt+sg4zt+sds.

Calculating the derivative of *V*_3_(*t*) along solutions of model (2), we obtain

(35) dV3tdt=sx1−fx2,v2fx,v2+fx,vfx2,v2fx,v2−fx2,v2g2vg2v2+M1+M2,

where

(36) M1=pem1τ1g1y2g4z−pbem1τ1cg4z=pem1τ1g4zg1y2−g1y4¯,M2=fx2,v2ln⁡g2vfx2,v2g2v2fx,v−fx2,v2·Hg2v2g1yt−τ2g1y2g2v−fx2,v2·Hg1y2fxt−τ1,vt−τ1g1yfx2,v2.

Therefore,

(37) dV3tdt=sx−sx21−fx2,v2fx,v2−fx2,v2Hg2v2g1yt−τ2g1y2g2v+fx2,v2g2vg2v2fx,vfx,v2−1·g2v2g2v−fx,v2fx,v+pem1τ1g4z·g1y2−g1y4¯−fx2,v2·Hg2vfx,v2g2v2fx,v−fx2,v2·Hg1y2fxt−τ1,vt−τ1g1yfx2,v2−fx2,v2Hfx2,v2fx,v2.

Note that (*s*(*x*) − *s*(*x*_2_))(1 − *f*(*x*_2_, *v*_2_)/*f*(*x*, *v*_2_)) ≤ 0, and

(38) fx,vfx,v2−1g2v2g2v−fx,v2fx,v≤0for  t≥0.

Since $y_{2}\leq \bar{y_{4}}$, we have *dV*_3_(*t*)/*dt* ≤ 0, and *dV*_3_(*t*)/*dt* = 0 if and only if *x*(*t*) = *x*_2_, *y*(*t*) = *y*_2_, *v*(*t*) = *v*_2_, and *z*(*t*) = 0. From LaSalle's invariance principle [36], we finally have that *E*_2_ is globally asymptotically stable when *R*_1_ > 1 and *R*_3_ ≤ 1.

Next, consider conclusion (b). By computing, the characteristic equation of linearization system of model (2) at *E*_2_ is

(39) $$\begin{array}{c} f_{1}\left(\;\lambda \;\right)f_{2}\left(\;\lambda \;\right)=0, \end{array}$$

where *f*_1_(*λ*) = *λ* + *b* − *ce* ^−*λτ*_3_^ *g*_1_(*y*_2_) and

(40) $$\begin{array}{c} f_{2}\left(\;\lambda \;\right)=\left|\begin{array}{cccc} a_{11} & 0 & a_{13} & 0\\ a_{21} & a_{22} & a_{23} & 0\\ 0 & a_{32} & a_{33} & a_{34}\\ 0 & 0 & a_{43} & a_{44} \end{array}\right|, \end{array}$$

where

(41) a11=λ−s′x2+∂fx2,v2∂x,a13=∂fx2,v2∂v,a21=−e−m1+λτ1∂fx2,v2∂x,a22=λ+ag1′y2,a23=−e−m1+λτ1∂fx2,v2∂v,a32=−ke−m2+λτ2g1′y2,a33=λ+u+qg3w2g2′v2,a34=qg2v2g3′w2,a43=−rg2′v2g3w2,a44=λ+h−rg2v2g3′w2.

When *R*_3_ > 1, we have $f_{1}(0)=b-cg_{1}(y_{2})=c(g_{1}(\bar{y_{4}})-g_{1}(y_{2}))<0$ and lim_*λ*→+*∞*_⁡ *f*_1_(*λ*) = +*∞*. Hence, there is also a positive root *λ* ^*∗*^ such that *f*_1_(*λ* ^*∗*^) = 0. Therefore, when *R*_3_ > 1, *E*_2_ is unstable. This completes the proof.

**Remark 11 .**

Theorem 10 shows that if only equilibria *E*_0_, *E*_1_, and *E*_2_ exist, then when *R*_3_ ≤ 1 and *R*_1_ > 1, *E*_2_ is globally asymptotically stable, and delays *τ*_1_, *τ*_2_, and *τ*_3_ do not impact the stability of *E*_2_.

### 3.4. Stability of Equilibrium *E*_3_

On the stability analysis of equilibrium *E*_3_, we only discuss the following case: *τ*_1_ ≥ 0, *τ*_2_ ≥ 0, and *τ*_3_ = 0. Other cases, *τ*_1_ ≥ 0, *τ*_2_ ≥ 0, and *τ*_3_ ≥ 0, are numerically verified for bifurcation phenomena and stability switches of *E*_3_ but the analytic analysis is left as an open problem. Before the proof of theorem, we have the following Lemma.

**Lemma 12 .**

Suppose *R*_2_ > 1 and *R*_4_ ≤ 1. Let $\bar{E_{4}}=(\bar{x_{4}},\bar{y_{4}},\bar{v_{4}},\bar{z_{4}},\bar{w_{4}})$ be the solution of (4) with $\bar{v_{4}}=g_{2}^{-1}(h/r)$ and $\bar{y_{4}}=g_{1}^{-1}(b/c).$ Then for equilibrium *E*_3_ = (*x*_3_, *y*_3_, *v*_3_, *z*_3_, 0), $v_{3}\leq \bar{v_{4}}$.

**Proof**

Since $\bar{E_{4}}$ satisfies (4), we have $\bar{y_{4}}=g_{1}^{-1}(b/c)$, $\bar{v_{4}}=g_{2}^{-1}(h/r)$, and $\bar{x_{4}}=x_{2}.$ Compared with *E*_4_, we get $\bar{y_{4}}=y_{4}$ and $\bar{v_{4}}=v_{4}.$ When *R*_4_ ≤ 1, we obtain $\bar{w_{4}}<0.$ Since

(42) ke−m2τ2g1y3=ug2v3,ke−m2τ2g1y4¯=ug2v4¯+qg2v4¯g3w4¯,

it follows that $v_{3}\leq \bar{v_{4}}$ if *R*_2_ > 1 and *R*_4_ ≤ 1. This completes the proof.

**Theorem 13 .**

Let *R*_2_ > 1. (a) If *R*_4_ ≤ 1 and *τ*_3_ = 0, then infection equilibrium *E*_3_ with only CTL response is globally asymptotically stable.

(b) If *R*_4_ > 1, then *E*_3_ is unstable.

**Proof**

We first consider conclusion (a). Define a Lyapunov functional *V*_4_(*t*) as follows:

(43) V4t=xt−∫x3xtfx3,v3fθ,v3 dθ+em1τ1yt−∫y3ytg1y3g1θ dθ+fx3,v3g2v3uvt−∫v3vtg2v3g2θ dθ+pem1τ1czt−∫z3ztg4z3g4θ dθ+qfx3,v3g2v3ruwt+fx3,v3∫−τ10Hfxt+s,vt+sfx3,v3ds+fx3,v3∫−τ20Hg1yt+sg1y3ds.

Calculating the derivative of *V*_4_(*t*) along solutions of model (2), we obtain that

(44) dV4tdt=sx1−fx3,v3fx,v3+fx,vfx3,v3fx,v3−fx3,v3g2vg2v3+qfx3,v3ug2v3g3w·g2v3−g2v4¯−fx3,v3·Hg2v3g1yt−τ2g1y3g2v+fx3,v3·ln⁡g2vfx3,v3g2v3fx,v−fx3,v3·Hg1y3fxt−τ1,vt−τ1g1yfx3,v3=sx−sx31−fx3,v3fx,v3−fx3,v3·Hg2v3g1yt−τ2g1y3g2v+fx3,v3·g2vg2v3fx,vfx,v3−1g2v3g2v−fx,v3fx,v−fx3,v3Hfx3,v3fx,v3−fx3,v3·Hg1y3fxt−τ1,vt−τ1g1yfx3,v3−fx3,v3Hg2vfx,v3g2v3fx,v+qfx3,v3ug2v3·g3wg2v3−g2v4¯.

Note that (*s*(*x*) − *s*(*x*_3_))(1 − *f*(*x*_3_, *v*_3_)/*f*(*x*, *v*_3_)) ≤ 0, and

(45) fx,vfx,v3−1g2v3g2v−fx,v3fx,v≤0for  t≥0.

Since $v_{3}\leq \bar{v_{4}}$, we have *dV*_4_(*t*)/*dt* ≤ 0, and *dV*_4_(*t*)/*dt* = 0 if and only if *x*(*t*) = *x*_3_, *y*(*t*) = *y*_3_, *v*(*t*) = *v*_3_, and w(*t*) = 0. From LaSalle's invariance principle [36], we finally have that *E*_3_ is globally asymptotically stable when *τ*_3_ = 0, *R*_0_ > 1, *R*_2_ > 1, and *R*_4_ ≤ 1.

Next, we consider conclusion (b). By computing, the characteristic equation of the linearization system of model (2) at *E*_3_ is

(46) $$\begin{array}{c} \left(\;\lambda +h-rg_{2}\left(\;v_{3}\;\right)\;\right)f\left(\;\lambda \;\right)=0, \end{array}$$

where

(47) $$\begin{array}{c} f\left(\;\lambda \;\right)=\left|\begin{array}{cccc} a_{11} & 0 & a_{13} & 0\\ a_{21} & a_{22} & a_{23} & a_{24}\\ 0 & a_{32} & a_{33} & 0\\ 0 & a_{42} & 0 & a_{44} \end{array}\right|, \end{array}$$

where

(48) a11=λ−s′x3+∂fx3,v3∂x,a13=∂fx3,v3∂v,a21=−e−m1+λτ1∂fx3,v3∂x,a22=λ+a+pg4z3g1′y3,a23=−e−m1+λτ1∂fx3,v3∂v,a24=pg1y3g4′z3,a32=−ke−m2+λτ2g1′y3,a33=λ+ug2′v3,a42=−ce−λτ3g4z3g1′y3,a44=λ+b−cg1y3e−λτ3g4′z3.

When *R*_4_ > 1, we have $h-rg_{2}(v_{3})=r(g_{2}(\bar{v_{4}})-g_{2}(v_{3}))<0$. Hence, there is a positive root *λ* ^*∗*^ = *rg*_2_(*v*_3_) − *h*. Therefore, when *R*_4_ > 1, *E*_3_ is unstable for any *τ*_1_ ≥ 0, *τ*_2_ ≥ 0, and *τ*_3_ ≥ 0. This completes the proof.

**Remark 14 .**

Theorem 13 shows that if only equilibria *E*_0_, *E*_1_, *E*_2_, and *E*_3_ exist, then when *R*_2_ > 1, *R*_4_ ≤ 1, and *τ*_3_ = 0, *E*_3_ is globally asymptotically stable, and delays *τ*_1_ and *τ*_2_ do not impact the stability of *E*_3_.

### 3.5. Stability of Equilibrium *E*_4_

On the stability analysis of equilibrium *E*_4_, we here only discuss the following case: *τ*_1_ ≥ 0, *τ*_2_ ≥ 0, and *τ*_3_ = 0. However, for the cases *τ*_1_ ≥ 0, *τ*_2_ ≥ 0, and *τ*_3_ ≥ 0, the theoretical analysis is very complicated. We will give numerical analysis for this case in the next section.

**Theorem 15 .**

If *τ*_3_ = 0, *R*_3_ > 1, and *R*_4_ > 1, then infection equilibrium *E*_4_ with both antibody and CTL immune responses is globally asymptotically stable.

**Proof**

Define a Lyapunov functional *V*_5_(*t*) as follows:

(49) V5t=xt−∫x4xtfx4,v4fθ,v4 dθ+em1τ1yt−∫y4ytg1y4g1θ dθ+fx4,v4g2v4u+qg3w4vt−∫v4vtg2v4g2θ dθ+pem1τ1czt−∫z4ztg4z4g4θ dθ+fx4,v4·∫−τ20Hg1yt+sg1y4ds+qfx4,v4g2v4u+qg3w4wt−∫w4wtg3w4g3θ dθ+fx4,v4·∫−τ10Hfxt+s,vt+sfx4,v4ds.

Using the above similar method, we obtain

(50) dV5tdt=sx−sx41−fx4,v4fx,v4−fx4,v4Hfx4,v4fx,v4+Hg2vfx,v4g2v4fx,v+Hg1y4fxt−τ1,vt−τ1g1yfx4,v4+Hg2v4g1yt−τ2g1y4g2v+fx4,v4·g2vg2v4fx,vfx,v4−1g2v4g2v−fx,v4fx,v.

Note that (*s*(*x*) − *s*(*x*_4_))(1 − *f*(*x*_4_, *v*_4_)/*f*(*x*, *v*_4_)) ≤ 0, and

(51) fx,vfx,v4−1g2v4g2v−fx,v4fx,v≤0for  t≥0.

Obviously, we have *dV*_5_(*t*)/*dt* ≤ 0, and *dV*_5_(*t*)/*dt* = 0 if and only if *x*(*t*) = *x*_4_, *y*(*t*) = *y*_4_, and *v*(*t*) = *v*_4_. From LaSalle's invariance principle [36], we finally have that *E*_4_ is globally asymptotically stable when *τ*_3_ = 0, *R*_3_ > 1, and *R*_4_ > 1. This completes the proof.

**Remark 16 .**

Theorem 15 shows that if equilibria *E*_0_, *E*_1_, *E*_2_, *E*_3_, and *E*_4_ exist, then when *R*_3_ > 1, *R*_4_ > 1, and *τ*_3_ = 0, *E*_4_ is globally asymptotically stable, and delays *τ*_1_ and *τ*_2_ do not impact the stability of *E*_4_.

## 4. Numerical Simulations

In the above section, we obtain the global asymptotic stability of equilibria *E*_3_ and *E*_4_ when the delay *τ*_3_ = 0. In this section, by using the numerical simulation, it is shown that the Hopf bifurcation and stability switches occur at equilibria *E*_3_ and *E*_4_ in the case *τ*_3_ > 0.

**Example 17 .**

**Figure 1:**
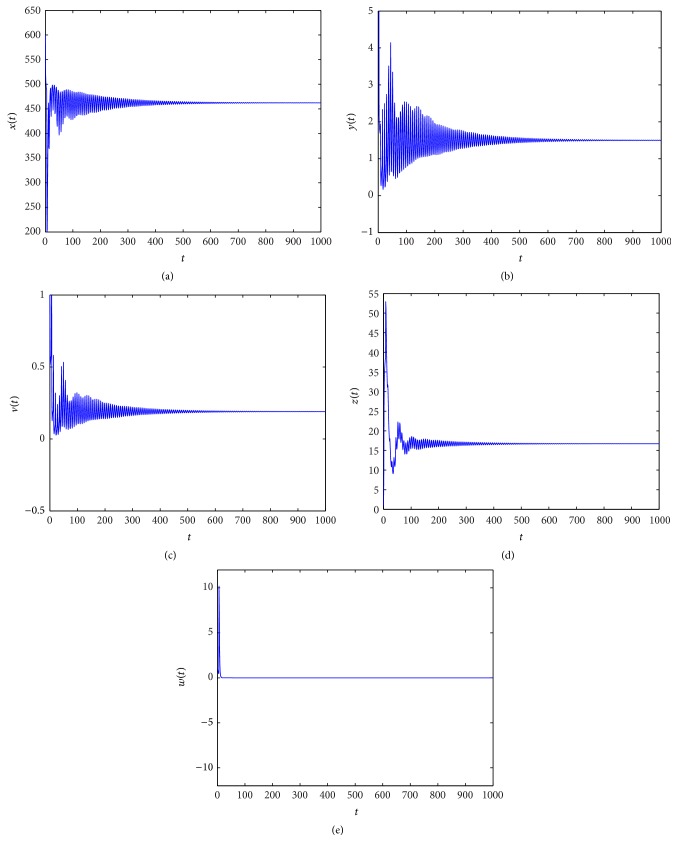
Taking *τ*_3_ = 0.2, we have *R*_2_ = 34.4139 > 1 and *R*_4_ = 0.2854 < 1, and the infection equilibrium *E*_3_ with only CTL response is asymptotically stable.

**Figure 2 fig2:**
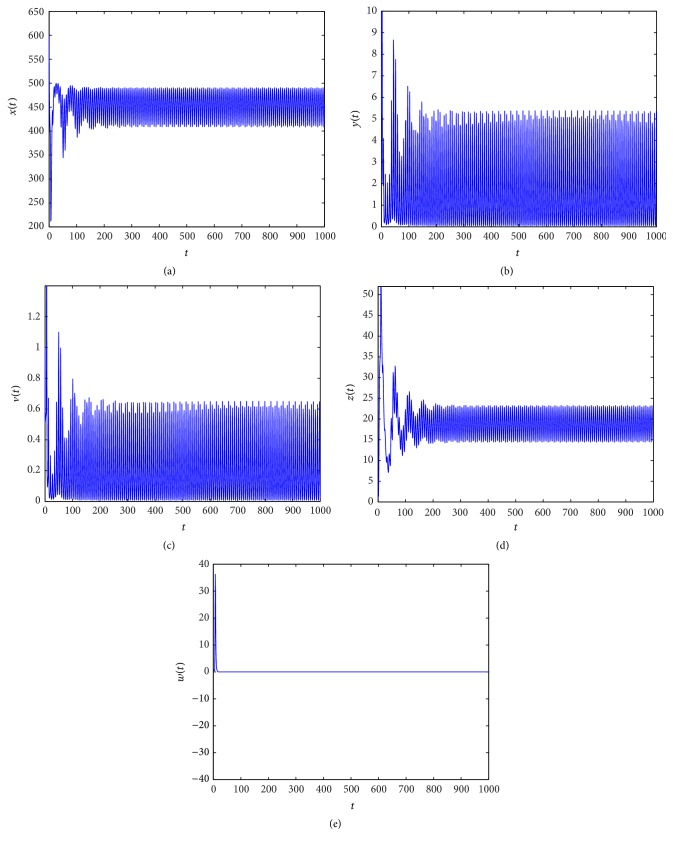
Taking *τ*_3_ = 2, we have *R*_2_ = 34.4139 > 1 and the Hopf bifurcation at infection equilibrium *E*_3_ with only CTL response occurs.

**Figure 3 fig3:**
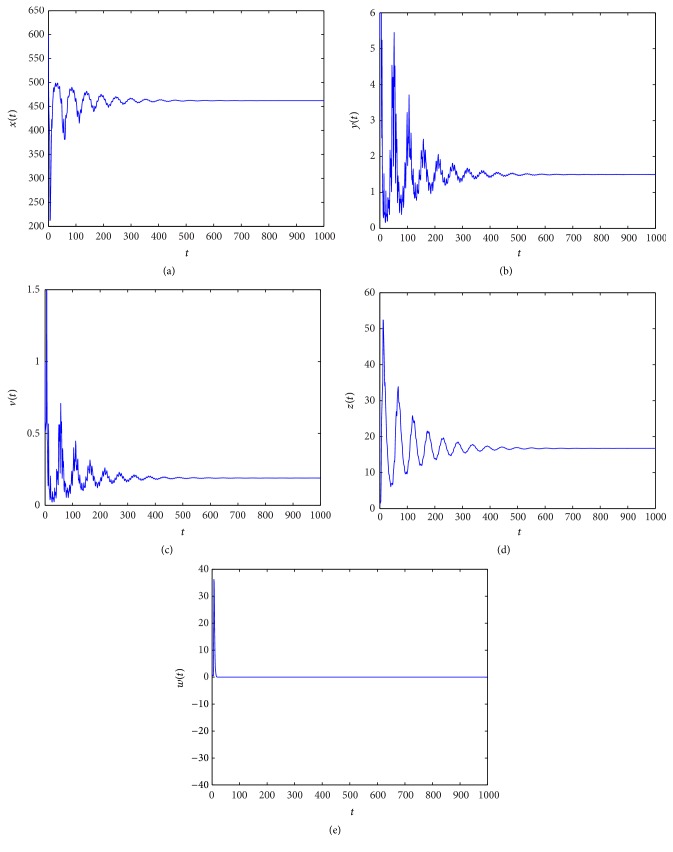
Taking *τ*_3_ = 4, we have *R*_2_ = 34.4139 > 1 and *R*_4_ = 0.2854 < 1, and the infection equilibrium *E*_3_ with only CTL response is asymptotically stable.

**Figure 4 fig4:**
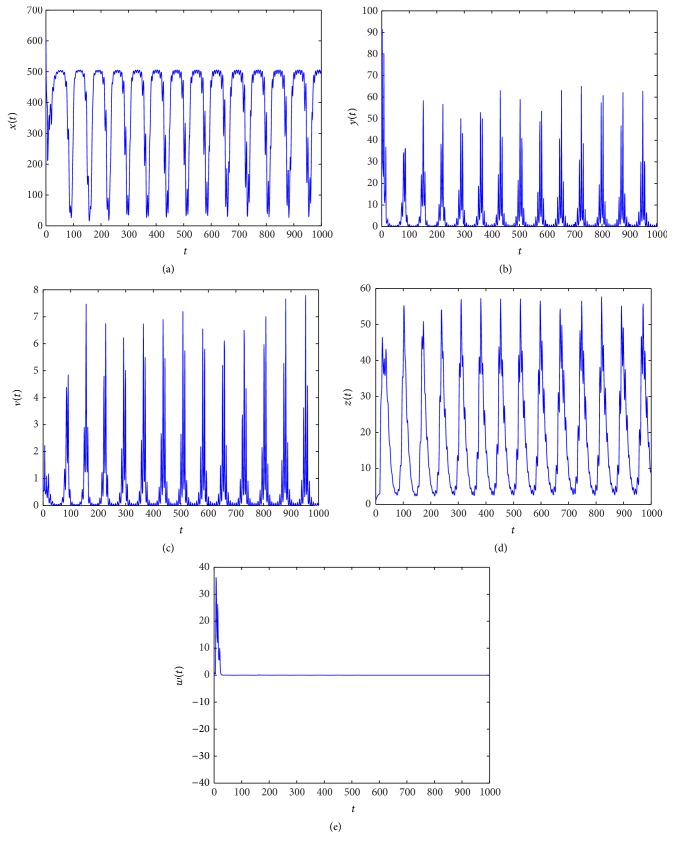
Taking *τ*_3_ = 15, we have *R*_2_ = 34.4139 > 1 and the Hopf bifurcation at infection equilibrium *E*_3_ with only CTL response occurs.

Corresponding to model (2), we consider the following model:

(52) dxtdt=λ−dxt+r1x1−xK−βxtvt−b1e−c1vt+b1,dytdt=βe−m1τ1xt−τ1vt−τ1−b1e−c1vt−τ1+b1−ayt−pytzt,dvtdt=ke−m2τ2yt−τ2−uvt−qvtwt,dztdt=cyt−τ3zt−τ3−bzt,dwtdt=rvtwt−hwt,

where *b*_1_, *c*_1_ > 0 are constants. We have *s*(*x*) = *λ* − *dx*(*t*) + *r*_1_ *x*(1 − *x*/*K*), *f*(*x*, *v*) = *βx*(*t*)((*v*(*t*) − *b*_1_)*e* ^−*c*_1_*v*(*t*)^ + *b*_1_), and *g*_i_(*ξ*) = *ξ*  (*i* = 1,2, 3,4). It can easily verify that (*H*_1_)–(*H*_4_) hold. Taking *λ* = 10, *d* = 0.01, *r*_1_ = 0.6, *K* = 500, *β* = 0.3, *c*_1_ = 0.01, *b*_1_ = 0.01, *a* = 0.5, *p* = 1, *k* = 0.4, *u* = 3, *q* = 1, *c* = 0.1, *b* = 0.15, *m*_1_ = *m*_2_ = 0.01, *g* = 1.5, *h* = 1, *τ*_1_ = 2, and *τ*_2_ = 5, choose *τ*_3_ as free parameter. By computing, *R*_2_ = 34.4139 > 1, *R*_4_ = 0.2854 < 1, and *E*_3_ = (462.1965,1.5000,0.1902,15.3959,0). From Figures 1 [Fig fig2] [Fig fig3]–4, we see that as *τ*_3_ increases the complex dynamical behaviors of equilibrium *E*_3_ occur.

In Figures 1 [Fig fig2] [Fig fig3] [Fig fig4] [Fig fig5] [Fig fig6] [Fig fig7]–8, we denote by (a) the time-series of *x*(*t*), by (b) the time-series of *y*(*t*), by (c) the time-series of *v*(*t*), by (d) the time-series of *z*(*t*), and by (e) the time-series of *w*(*t*).

**Example 18 .**

Corresponding to model (2), we consider the following model:

(53) dxtdt=λ−dxt−βxtvt1+a1xt+b1vt+a1b1xtvt,dytdt=βe−m1τ1xt−τ1vt−τ11+a1xt−τ1+b1vt−τ1+a1b1xt−τ1vt−τ1−ayt−pytzt,dvtdt=ke−m2τ2yt−τ2−uvt−qvtwt,dztdt=cyt−τ3zt−τ3−bzt,dwtdt=rvtwt−hwt,

where *a*_1_, *b*_1_ > 0 are constants. We have *s*(*x*) = *λ* − *dx*(*t*), *f*(*x*, *v*) = *βx*(*t*)*v*(*t*)/(1 + *a*_1_ *x*(*t*) + *b*_1_ *v*(*t*) + *a*_1_ *b*_1_ *x*(*t*)*v*(*t*)), and *g*_*i*_(*ξ*) = *ξ*  (*i* = 1,2, 3,4). It is easily verified that (*H*_1_)–(*H*_4_) hold.

**Figure 5 fig5:**
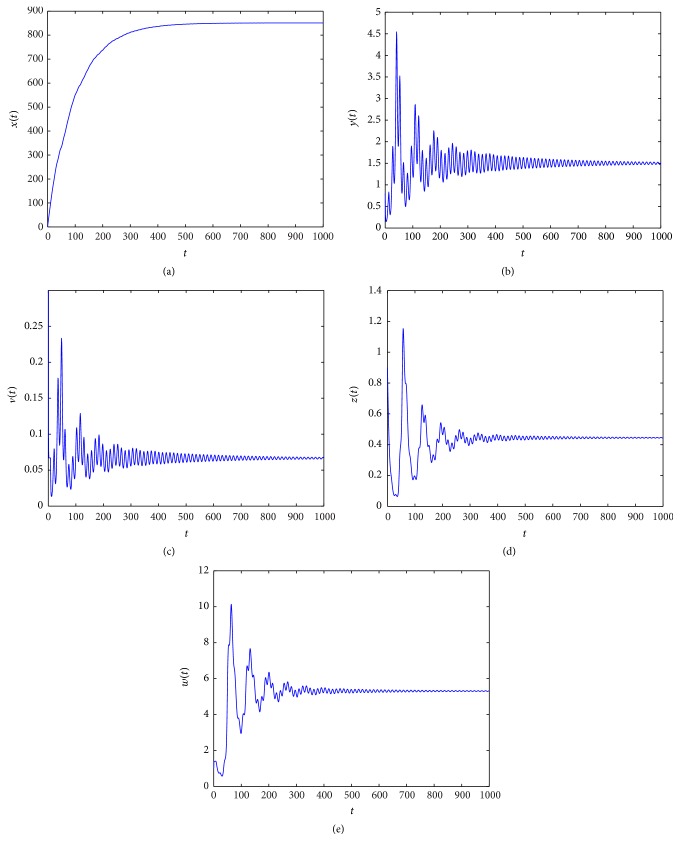
Taking *τ*_3_ = 0.1, we have *R*_3_ = 1.8912 > 1 and *R*_4_ = 2.7693 > 1, and the infection equilibrium *E*_4_ with both CTL and antibody responses is asymptotically stable.

**Figure 6 fig6:**
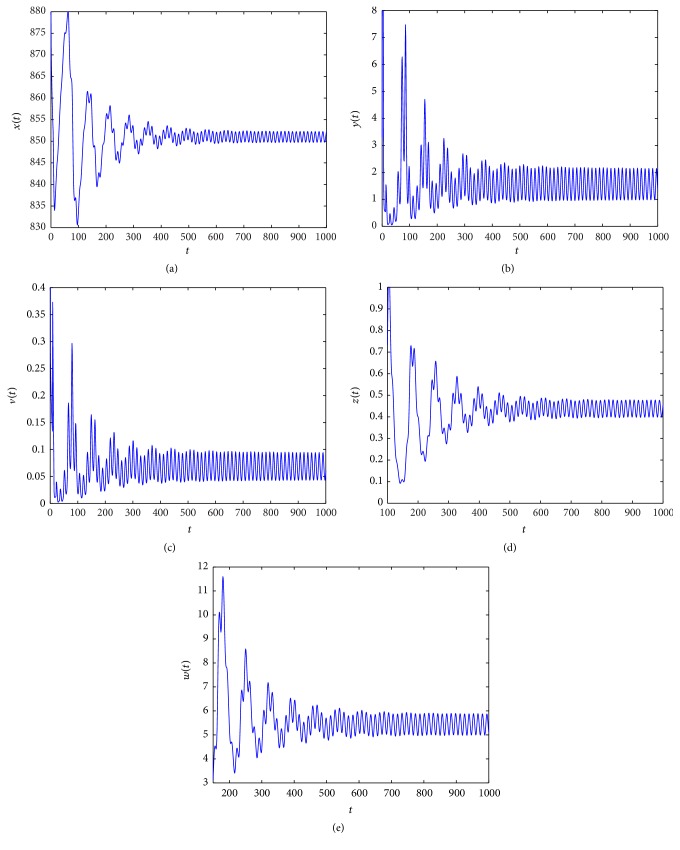
Taking *τ*_3_ = 2.5, we have *R*_3_ = 1.8912 > 1 and *R*_4_ = 2.7693 > 1, and the Hopf bifurcation at infection equilibrium *E*_4_ with both CTL and antibody responses occurs.

**Figure 7 fig7:**
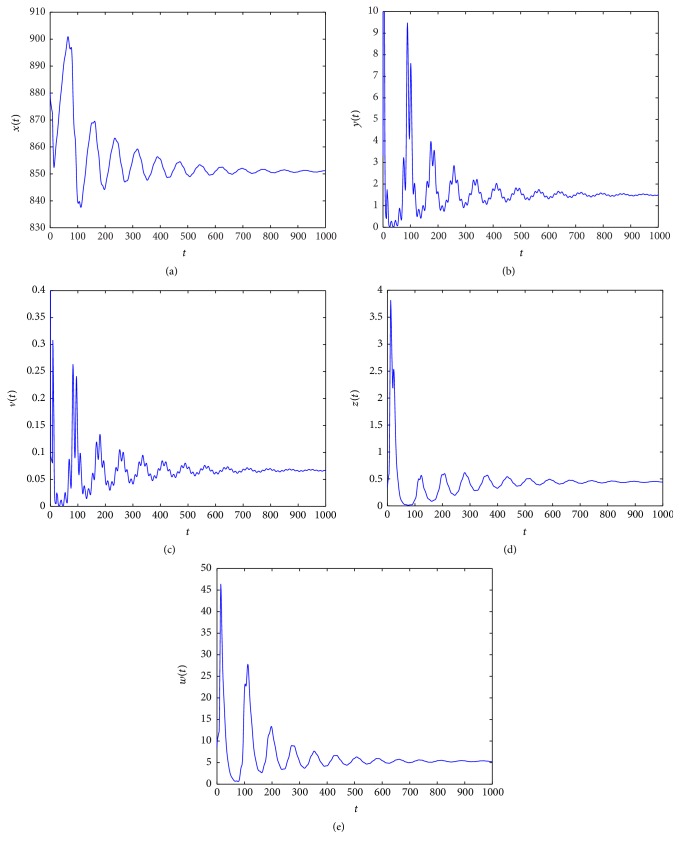
Taking *τ*_3_ = 6, we have *R*_3_ = 1.8912 > 1 and *R*_4_ = 2.7693 > 1, and the infection equilibrium *E*_4_ with both CTL and antibody responses is asymptotically stable.

**Figure 8:**
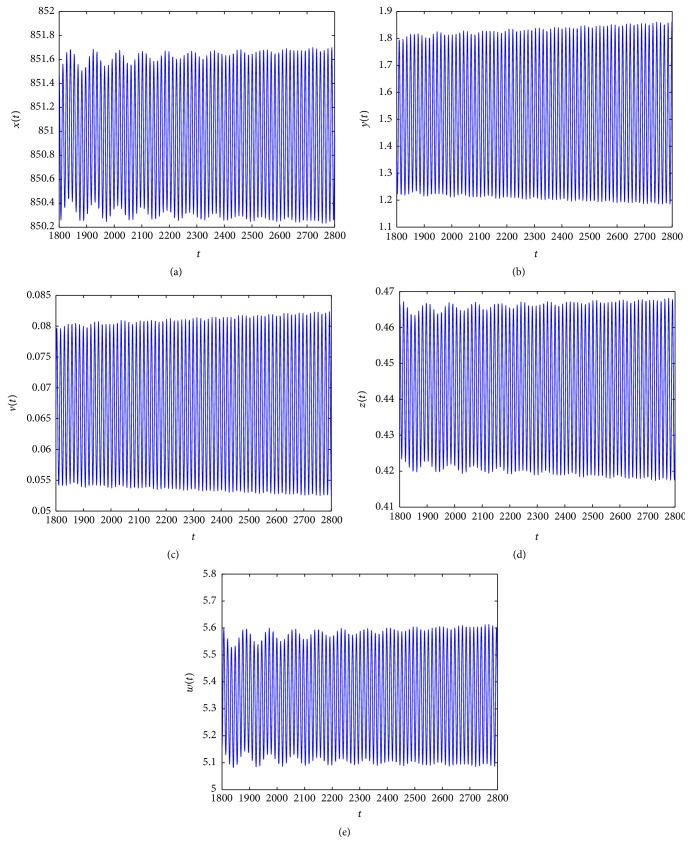
Taking *τ*_3_ = 16, we have *R*_3_ = 1.8912 > 1 and the Hopf bifurcation at infection equilibrium *E*_4_ with both CTL and antibody responses occurs.

Taking *λ* = 10, *d* = 0.01, *β* = 0.25, *a*_1_ = 0.01, *b*_1_ = 0.01, *a* = 0.5, *p* = 1, *k* = 0.4, *u* = 3, *q* = 1, *c* = 0.1, *b* = 0.15, *m*_1_ = *m*_2_ = 0.01, *r* = 1.5, *h* = 0.1, *τ*_1_ = 5, and *τ*_2_ = 8, choose *τ*_3_ as free parameter. By computing, *R*_3_ = 1.8912 > 1, *R*_4_ = 2.7693 > 1, and *E*_4_ = (850.8857,1.5000,0.6667,0.4456,5.3039). From Figures 5 [Fig fig6] [Fig fig7]–8, we see that as *τ*_3_ increases the complex dynamical behaviors of equilibrium *E*_4_ occur.

## 5. Discussion

In this paper we have considered an in-host model with intracellular delay *τ*_1_, virus replication delay *τ*_2_, and immune response delay *τ*_3_, given by (2) together with assumptions (*H*_1_)–(*H*_4_), which describes the dynamics among uninfected cells, infected cells, virus, CTL responses, and antibody responses. The model allows for general target-cell dynamics *s*(*x*), including a nonlinear incidence *f*(*x*, *v*), discrete delays, and state-dependent removal functions *g*_*i*_  (*i* = 1,2, 3,4). This general model includes many existing models in the literature as special cases. Dynamical analysis of model (2) shows that *τ*_1_, *τ*_2_, and *τ*_3_ play different roles in the stability of the equilibria. Particularly, we see that *τ*_3_ may impact the stability of equilibria *E*_3_ and *E*_4_.

By the analysis, we have shown that when *R*_0_ ≤ 1, *E*_0_ is globally asymptotically stable, which means that the virus is cleared up. When *R*_0_ > 1, *R*_1_ ≤ 1, and *R*_2_ ≤ 1, *E*_1_ is globally asymptotically stable, which means that the infection is successful, but the establishments of both antibody and CTLs immune responses are unsuccessful. When *R*_1_ > 1 and *R*_3_ ≤ 1, *E*_2_ is globally asymptotically stable, which implies that the antibody response is established, but the infected cells are too weak to stimulate CTL immune response. With respect to the analysis of *E*_3_, we consider special cases *τ*_3_ = 0, *τ*_1_ ≥ 0, and *τ*_2_ ≥ 0; when *R*_2_ > 1 and *R*_4_ ≤ 1, *E*_3_ is globally asymptotically stable, which means that the CTL immune response is determined, but the viral loads are so small that it cannot activate the antibody response. About the stability of *E*_4_, we have obtained that for special case, *τ*_3_ = 0, *τ*_1_ ≥ 0, and *τ*_2_ ≥ 0, when *R*_3_ > 1 and *R*_4_ > 1, *E*_4_ is globally asymptotically stable, that is, susceptible cells, infected cells, free virus, CTLs, and antibodies coexist in vivo.

Based on Theorems 13 and 15, we obtain that the intracellular delay *τ*_1_ and virus replication delay *τ*_2_ for model (2) do not cause Hopf bifurcation. Moreover, *R*_0_ plays a crucial role in virus infection dynamics. Actually, in model (2), *R*_0_ is a decreasing function on time delay *τ*_1_. When all other parameters are fixed and delay *τ*_1_ is sufficiently large, *R*_0_ becomes less than one, only infection-free equilibrium *E*_0_ exists, and the virus is cleared in the host. By biological meanings, intracellular delay plays a positive role in virus infection process in order to eliminate virus. Sufficiently large intracellular delay makes the virus development slower and the virus has been controlled and disappeared. This gives us some suggestions on new drugs to prolong the time of infected cells producing virus. However, by the recent research of Li and Shu [37], in the case of the coexistence of mitosis rate of the target cells and an intracellular delay in the viral infection model, the intracellular delay produces Hopf bifurcation only when the mitosis rate is sufficiently large.

When *τ*_3_ > 0, by numerical simulations, it is shown that the Hopf bifurcation and stability switches occur at equilibria *E*_3_ and *E*_4_ as *τ*_3_ increases. Figures 1 [Fig fig2] [Fig fig3]–4 indicate that *E*_3_ remains stable as *τ*_3_ > 0 is small, and along with the increase of *τ*_3_, equilibrium *E*_3_ becomes unstable and periodic oscillations appear. It shows that stability switches occur as delay *τ*_3_ increases. Similarly, from Figures 5 [Fig fig6] [Fig fig7]–8, we see that along with the increases of *τ*_3_ > 0 the dynamical behaviors of model (53) at equilibrium *E*_4_ appear as very large diversification. Particularly, when *τ*_3_ is small enough, *E*_4_ is asymptotically stable and when *τ*_3_ is increasing, the stability switches occur at equilibrium *E*_4_, and when *E*_4_ is unstable, a Hopf bifurcation occurs. Finally, when *τ*_3_ is enough large, equilibrium *E*_4_ always is unstable. Summarizing these discussions, we have the conclusion that *τ*_3_ affects markedly the stability of equilibria *E*_3_ and *E*_4_. From the numerical simulations, we observe that immune response delay *τ*_3_ can cause Hopf bifurcation. Upon primary infection, the sustained oscillations from the Hopf bifurcation imply that the pathogen may not always be cleared entirely with the CTL responses which usually occur in a few days after serum conversion. As the increase of immune delay *τ*_3_, we know that the drug prevents virus from continuing through their cell cycle, thus trapping them at some point during interphase, where the cells die from natural causes. Then susceptible cells, infected cells, free virus, CTLs, and antibodies reach a stable level in the host. When immune delay *τ*_3_ continuously increases, the activation of the immune cell is to fight against the malignant virus cells. Thus susceptible cells, infected cells, free virus, CTLs, and antibodies exhibit sustained periodic oscillations in the chronic phase of infection. This explains the fact that the immune response delay plays negative roles in controlling disease progression.

Observing all obtained results in this paper, we can directly put forward the following open questions which need to be further studied in the future.

For one, in addition to *τ*_1_, *τ*_2_, and *τ*_3_, antibody response delay *τ*_4_ is also considered, whether the results obtained in this paper can be extended to a virus infection model with nonlinear incidence rate and four time delays. For another, we obtain the Hopf bifurcation and stability switches at equilibria *E*_3_ and *E*_4_ for model (2) only by using the numerical simulation method for special examples (52) and (53). Up to now, the theoretical analysis and results in this aspect are few and rough. Therefore, a systemic and complete theoretical analysis and results will be a very estimable and significative subject.
